# Migration and Removal of Labile Cadmium Contaminants in Paddy Soils by Electrokinetic Remediation without Changing Soil pH

**DOI:** 10.3390/ijerph19073812

**Published:** 2022-03-23

**Authors:** Yajun Luan, Junzeng Xu, Jing Zhou, Haiyu Wang, Fengxiang Han, Kechun Wang, Yuping Lv

**Affiliations:** 1 State Key Laboratory of Hydrology-Water Resources and Hydraulic Engineering, Hohai University, Nanjing 210098, China; luan450705@163.com (Y.L.); rohana0218@163.com (J.Z.); haiyuwang@163.com (H.W.); kechunw101@hhu.edu.cn (K.W.); 2 College of Agricultural Science and Engineering, Hohai University, Nanjing 210098, China; 3 Department of Chemistry and Biochemistry, Jackson State University, Jackson, MS 39217, USA; fengxiang.han@jsums.edu; 4 College of Hydraulic Science and Engineering, Yangzhou University, Yangzhou 225009, China; lvyupingsun@126.com

**Keywords:** paddy soils, cadmium, electrokinetic remediation, polarity reversal, soil pH

## Abstract

Electrokinetic remediation (EKR) is a viable, advanced cleaning strategy that can permanently reduce the toxicity of soil contaminants. However, EKR is prone to causing changes in soil pH. The negative impacts must be minimized if field-scale application is to be realized. In this study, EKR with polarity reversal was used to avoid soil pH polarization and to clean up cadmium (Cd)-contaminated paddy soils. Results showed that Cd desorbed from oxidizable and residual fractions to labile and easily available parts. Soil moisture content above 0.35 g g^−1^ was conductive to achieving the desirable Cd-migration rate. The exchangeable Cd phase eventually migrated from both ends of that soil compartment towards the intermediate. Moreover, the addition of citric acid at the concentration of 0.1 mol L^−1^ was an effective enhancement strategy. The methodology enriched Cd contaminants to specific sites. The technology can be used for electrokinetic-assisted phytoremediation during the rice growing period. Hyperaccumulator is planted in the intermediate area to remove the Cd contaminants. On the other hand, Cd removal is achieved in the region close to the electrodes. The present study provides a theoretical basis for in situ remediation. It has a wider significance for field-scale application.

## 1. Introduction

Heavy metal pollution in agricultural soils has become a worldwide threat to human health and safe food production. It has attracted wide attention [1]. Heavy metal contamination in agricultural ecosystems originates from anthropogenic activities, such as fertilization and irrigation, and tends to accumulate over time [2,3]. Moreover, long-term fertilization or acid rain can reduce soil pH, which activates heavy metals in soil and increases its bioavailability, thereby posing threats to human health [4]. Thus, decontamination of contaminated soil and reduction in metal contaminants in grains are urgent demands.

Several remediation solutions have been developed to clean polluted soil and ensure grain security, including screening of low-accumulation cultivars [5,6], reducing the bioavailability of heavy metals in soils [7,8], and removing pollutants from soils through electrokinetic extraction, phytoremediation, bioremediation, or soil washing [9,10]. In situ chemical immobilization is a technique that has been widely considered by researchers [11]. However, this technique does not achieve the removal of contaminants from soil. Long-term stability of heavy metals in soils cannot be guaranteed. External influences, such as acid rain, can reactivate heavy metals. Thus, in situ contaminant removal or extraction remediation is more favorable. Removing pollutants from soils is a promising soil-cleaning strategy that permanently reduces the toxicity of soil contaminants and minimizes their entry into crops. Among them, electrokinetic remediation (EKR) is a viable and advanced technology that uses a direct current (DC) electric field to mobilize polluted ions towards the opposite-charged electrode. This technology has been widely used due to its high efficiency, and has achieved favorable results [12,13]. However, one of the disadvantages is that EKR is prone to cause changes in the physical and chemical properties of soil [14]. The negative impacts caused by EKR must be minimized if field-scale application is to be realized.

During EKR process, the electrolysis of water leads to the division of soil areas into acidic zone (H_2_O − 2e^−^→2H^+^ + 1/2O_2_) and alkaline zone (2H_2_O + 2e^−^→2OH^−^ + H_2_). The hydrogen (H^+^) ions from the oxidation of water make it easier to desorb metal cations from the soil surface. The hydroxyl (OH^−^) ions produced in the alkaline zone have a negative effect on metal transportation [10]. This raised two problems: soil acidification in the acidic zone and focusing precipitation in the alkaline zone. Soil acidification in the acidic zone disrupts the soil nutrient cycle. Low soil pH near the anode negatively affects plant growth [15,16,17]. Considering that the land would continue to be used for growing crops after remediation, soil acidification is one of the disadvantages that limits the practical application of EKR technology [18]. On the other hand, with the increase in soil pH in the alkaline area, the solubility of heavy metals decreases, and the migration of metal ions is limited [19]. Various methods have been developed to solve the focusing phenomenon during the EKR process. For example, using the appropriate conditioning solutions (HNO_3_, HCl et al.) at the cathode can help to control the pH, increasing the exchangeable fraction of metals and indicating better performance for pollutant removal [20,21]. By using ion exchange membranes, the influx of H^+^ and OH^−^ ions into soil can be impeded, this showed good results in preventing a wild pH variation and precipitation with metal ions in soil in [22]. These methodologies have shown good results in decreasing soil pH and improving metal solubility in the alkaline zone. However, chemical additives are not environmentally acceptable, and extra materials could be costly. Therefore, the EKR technology faces challenges in green and sustainable development, which requires more environmentally friendly and cost-effective methods for contaminated soil remediation [23,24].

Various biodegradable chelates have been found to combine with several kinds of heavy metals, and increase ion solubility, without requiring changes in soil pH [25,26]. Among these biodegradable chelates, low-molecular-weight organic acids, such as citric acid, oxalic acid, and acetic acid, are environmentally friendly for soil remediation, owing to their buffering, biodegradability, chelating, and complexing actions. Previous studies have shown that the supplied chloride ions from electrolyte would combine with heavy metals as a complexing agent, and then these metal complexes can increase the removal efficiency [27]. Other studies have also shown that citric acid can efficiently remove metals in electrokinetic remediation. Citric acid acts as a buffer substance, which quickly neutralizes the OH^−^ produced in the cathode. As a chelating agent, citric acid has also shown high removal efficiency for harbor sediments and electroplating sludge [28]. Many studies have proven that citric acid can efficiently remove metals in electrokinetic remediation. It helps to desorb metals and transfer them from the solid phase to the liquid phase [29,30]. Using citric acid could effectively improve the removal rate of the metals in the contaminated soil [31].

Periodic reversal of polarity during the EKR process is another effective method to avoid changes in soil pH. Metals were redissolved by the polarity exchange technique. Both the pH adjustment of the soil system and the control of the direction of contaminant migration could be achieved without additional costs. The application of this technique showed a removal of 72% of the initial manganese in 7.6 d in [32]. In field experiments, EKR with the polarity reversal operation could partially resolve the adverse effects of EKR on soil pH and remove chromium more efficiently [33]. Mao et al. [34] found that EKR enhanced with an electrode polarity inversion strategy was an effective technique for lead decontamination from a low-permeability matrix. The electrode reversal was conducted for around 48 h to avoid pH polarization and Pb precipitation.

Soil moisture content is critical in achieving effective removal of the pollutants under EKR. Electric current and pollutants removal efficiency are positively correlated to soil moisture content. After a 72 h EKR experiment, it was found that the removal rate of total chromium was 14.4% with a soil moisture of 0.20 g g^−1^, and the removal rate was 67.3% with a soil moisture of 0.40 g g^−1^ [35]. The moisture of the kaolinite specimen in the electrokinetic cell was adjusted from 0.40 g g^−1^ to 0.60 g g^−1^. The increase in the moisture content clearly favored the electromigration of manganese towards the cathode. The increase in the moisture content up to 0.60 g g^−1^ resulted in over 90% manganese removal [36]. On the other hand, as an efficient water-saving irrigation model, controlled irrigation (CI) is widely used in China [37,38]. There is almost no water layer during the growth period of rice under CI. The soil gravimetric water content mostly ranges from 0.29 g g^−1^ to 0.42 g g^−1^, which is near the soil moisture condition that has an efficient removal efficiency under EKR treatment [39,40]. Thus, placing the electrodes directly into the moist soil to obtain optimum performance in contaminated paddy soils under alternate wetting and drying (AWD) moisture conditions deserves further consideration. It was expected to guide the potential application for in situ remediation in paddy fields under CI irrigation.

Electrokinetic remediation as an in situ cleaning technology has attracted significant attention from researchers. The challenge of pH control has greatly limited the field application of EKR technology in Cd-contaminated paddy soils. In the present research, cadmium (Cd) was selected as the target toxic metal, which has been listed as a priority pollutant in soil contamination monitoring, with an over-standard rate of 7.0% according to the National Soil Pollution Survey Bulletin of China in 2014 [41,42]. The Cd-contaminated soil was taken from local paddy fields. Laboratory experiments were performed by inserting the electrodes vertically into the moist soils to drive the electromigration of heavy metals. A cycle progress of pulsed electric fields (10 h ON/14 h OFF) was conducted [43,44]. Three different voltage intensities were set to investigate the changes in electric current, soil pH, Cd fractions, and the migration rates of soil Cd. The objectives were as follows: (1) to study the mobilization and redistribution of Cd in soils under EKR with polarity reversal, and to explore the feasibility of mobilizing Cd in a specific area; (2) to study the effect of citric acid on the migration rate of Cd. The significance of this study lies in providing theoretical support for an innovative, in situ electrokinetic remediation, and in its exploration of a scientific approach to remove Cd from soils and hence reduce Cd content in rice.

## 2. Materials and Methods

### 2.1. Site Description

The experiment was conducted in 2020 at Kunshan Experiment Station (31°15′50″ N; 120°57′43″ E), Eastern China. The soil texture was sandy clay, in which sand accounted for 41.20%, silt accounted for 51.92%, and clay accounted for 6.88%. The saturated soil moisture content was 0.403 g g^−1^. Soil pH was 7.41, soil bulk density was 1.30 g cm^−3^, and the soil organic matter content was 25.82 g kg^−1^. The Cd concentration was 0.793 mg kg^−1^. According to Environmental Quality Standard for Soils in China (GB15618-2018), the Cd concentration was higher than the risk screening value (0.6 mg kg^−1^ for paddy fields, with soil pH ranging from 6.5 to 7.5), indicating an ecological risk [45].

### 2.2. Electrokinetic Experiment Setup

**Figure 1:**
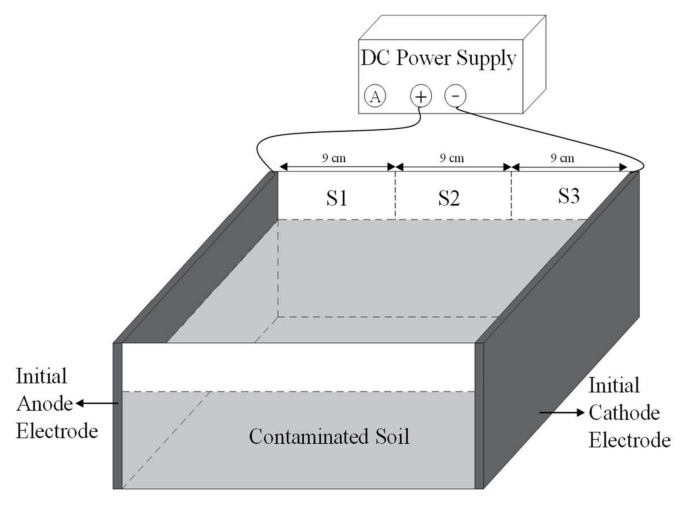
The electrokinetic remediation experiment diagram.

The EKR experimental diagram is shown in Figure 1. The experimental apparatus comprised three parts: the soil compartment, the graphite electrodes, and the DC power supply with an ammeter (DC power supply 305, Mestek, Shenzhen, China). The dimensions of the soil compartment were 40 cm × 29 cm × 20 cm (L × B × H). The soil used in the experiments was taken from the 0–20 cm soil layer of local paddy fields. After air-drying and crushing, the coarse fragments in the soil were removed with a 4 mm sieve. Then, the soil was packed into pots at a depth of 15 cm, with the bulk density of 1.3 g cm^−3^. Plate graphite electrodes (L × H × T: 40 cm × 18 cm × 1 cm) were used for the anode and cathode, respectively. Two graphite electrodes were inserted vertically into the contaminated soils at a net distance of 27 cm.

Table 1 summarizes the details of the experimental conditions. The experiment adopted the power supply of 1.0 V/cm, 0.8 V/cm, and 0.5 V/cm, namely EKR 1.0, EKR 0.8, and EKR 0.5, respectively. Each treatment was repeated three times. To match the soil moisture conditions of paddy fields under CI irrigation, soil gravimetric moisture content was controlled between 0.310 g g^−1^ and 0.403 g g^−1^. EKR experiments were run for two AWD cycles over 14 days. A cycle progress of pulsed electric fields (10 h ON/14 h OFF) was conducted in this research. One cycle was the sum of time with electric field in “ON” + “OFF”, that was 24 h. In total, there were 14 periodic cycles. The polarity reversal was operated after one cycle. A total of 14 reversal operations were performed in this study. As shown in Figure 1, the soil was sectioned into three equal parts, labeled as S1, S2, and S3, between the anode and the cathode. Soil samples were taken from each section using a 1 cm diameter U-shaped shovel, and were used to analyze soil pH and Cd concentration. Three soil samples were taken from each soil section, mixed well, and used for measuring. Soils were sampled when the pulsed system was off. The sampling times were 34 h, 130 h, 226 h, 274 h, and 322 h during the EKR process.

### 2.3. Citric Acid Preacidification Enhancement Experiment

Citric acid is a biodegradable organic acid, which is safe for the environment. In this study, it was used to enhance the removal of heavy metals. Other conditions were the same as other EKR experiments, except that we added citric acid into the contaminated soils before the experiments started. After filling the air-dried soil into the soil compartment, citric acid was added into the soils at a concentration of 0.1 mol L^−1^ so that the moisture content reached 0.3 g g^−1^. After that, the soil was saturated with deionized water. The concentration has been determined to preacidify the soil and enhance the electroosmotic flow [46,47]. The preacidified soil was equilibrated for 24 h before the DC system was powered on. The citric acid enhancement experiments (EKR^C^) were run for 1 AWD cycle over 6 d (Table 1). At the end of the EKR^C^ experiment, soil samples were taken from different sections to analyze soil pH and Cd concentrations.

### 2.4. Analysis and Calculation

The soil moisture content was calculated by weight loss after heating in an oven for 8 h at 105 °C [48]. The soil pH was measured using the pH meter (Mettler-Toledo, Zurich, Switzerland) by preparing slurries with a soil to water ratio of 1:2.5 [49]. The current was measured using the ammeter.

Metal speciation in the soil was determined through a sequential extraction method according to the procedure recommended by the Standards, Measurements, and Testing (former BCR) Program of the European Commission [50,51]. The metals were divided into exchangeable fraction (F1-Cd), reducible fraction (F2-Cd), and oxidizable fraction (F3-Cd). The residual (F4-Cd) was considered as the mineral non-extractable form. The metals ions concentration in the supernatant collected in different steps of the sequential extraction was filtered through a 0.45 μm membrane and analyzed by inductively coupled plasma optical emission spectrometry (ICP-OES, Thermo Scientific, Waltham, MA, USA).

The Cd migration rate, η, was calculated as follows:

(1) $$\eta =\frac{C_{t}-C_{0}}{C_{0}}\times 100\%$$

where $C_{t}$ was the Cd concentration at time, and $C_{0}$ was the initial Cd concentration.

The electrical energy consumption per unit mass ($E_{U}$) was calculated as follows:

(2) $$E_{U}=\frac{1}{m}\int_{0}^{t}UIdt$$

where $E_{U}$ was the electrical energy consumption per unit mass (kWh kg^−1^), *m* was the mass of migrated Cd (kg), *U* was the voltage between the electrodes (V), *I* was the electric current (A), and *t* was the power-on time (h).

### 2.5. Statistical Analysis

One-way ANOVA and the least significant difference (LSD) test for analysis with a significance level of *p* < 0.05 were used. Statistical analyses were performed using SPSS Statistics 24.

## 3. Results

### 3.1. Electric Current, Soil Moisture Content, and Soil pH Variation

**Figure 2:**
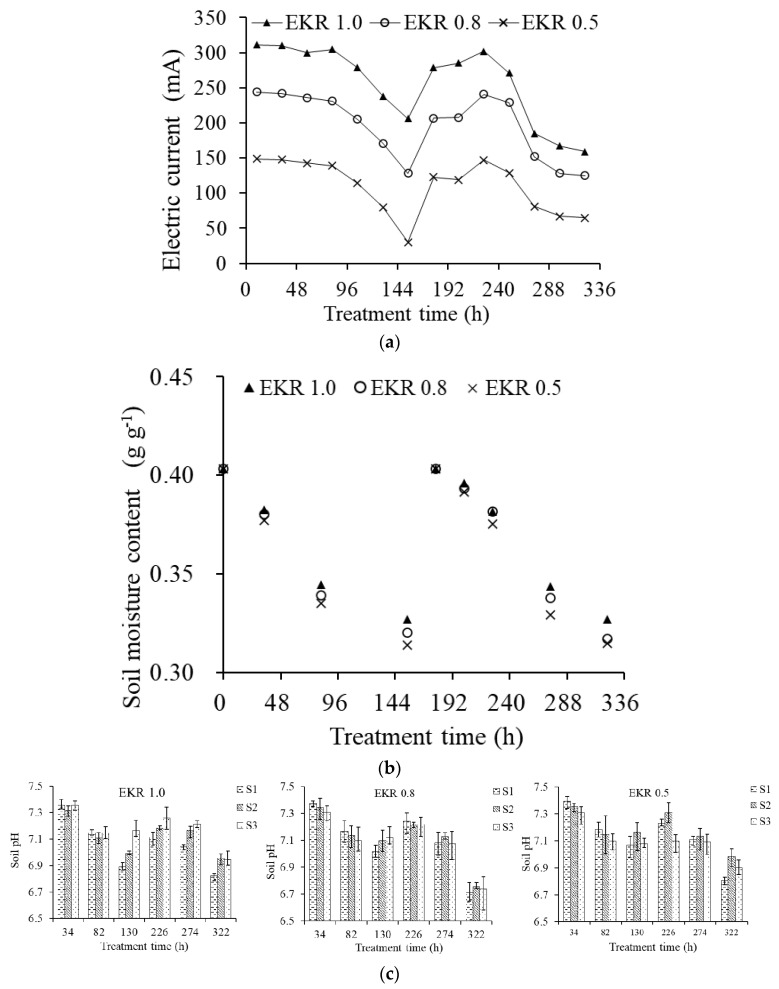
Electric current (**a**), soil moisture content variation (**b**), and soil pH in different soil sections (**c**) during the electrokinetic remediation process.

The electric current and soil moisture content at different voltage gradients during the EKR process are shown in Figure 2. The electric current was proportional to the voltage gradient (Figure 2a). The soil moisture content was similar among different treatments. The lower the voltage gradient, the slightly smaller the gravimetric moisture content (Figure 2b). The electric current was also proportional to the soil moisture content. The higher the gravimetric moisture content, the greater the current intensity. The current variation ranges were 206–311 mA, 128–244 mA, and 30–149 mA at different voltage gradients from 0 to 144 h, respectively, and were 159–302 mA, 125–241 mA, and 65–147 mA from 144 to 322 h. The electric current decreased significantly from 106 h to 154 h, as well as from 250 h to 322 h. Correspondingly, the soil gravimetric moisture content ranged from 0.321 g g^−1^ to 0.345 g g^−1^, and from 0.314 g g^−1^ to 0.357 g g^−1^ for those two time periods. Generally, the smaller the current, the lower the migration rate of Cd ions in the soil. Therefore, it can be assumed that, when the soil gravimetric moisture content was higher than 0.35 g g^−1^, the electric current was higher, and the metal ions could have a desirable migration efficiency, which was more favorable for the remediation of contaminated soil.

The soil pH was initially 7.41 and decreased gradually along with the reduction in soil moisture (Figure 2c). At 226 h, soil pH increased slightly due to the increase in soil moisture after irrigation. After 226 h, the pH continued to decrease. At the end of EKR process, soil pH of S1, S2, and S3 soil sections decreased by 0.59, 0.45, and 0.46 pH units, respectively, for EKR 1.0; the soil pH of S1, S2, and S3 soil sections decreased by 0.70, 0.65, and 0.67 pH units, respectively, for EKR 0.8; and the soil pH of S1, S2, and S3 soil sections decreased by 0.61, 0.43, and 0.51 pH units, respectively, for EKR 0.5. There were no significant differences in soil pH among the treatments. Generally, soil pH ranged from 6.71 to 7.41, basically maintained in the neutral. The soil itself possessed a certain buffer ability of protons. During the EKR process, the electrolysis of water released H^+^ and OH^−^ at the anode and cathode surface, respectively. H^+^ and OH^−^ produced by electrolysis reactions could be directly neutralized after the polarity reversal operation. The acidification effect on the soil was minimized, and the formation of alkaline environment was also avoided, which could reduce the precipitation of Cd.

### 3.2. Migration and Redistribution of Cd during EKR process

**Figure 3:**
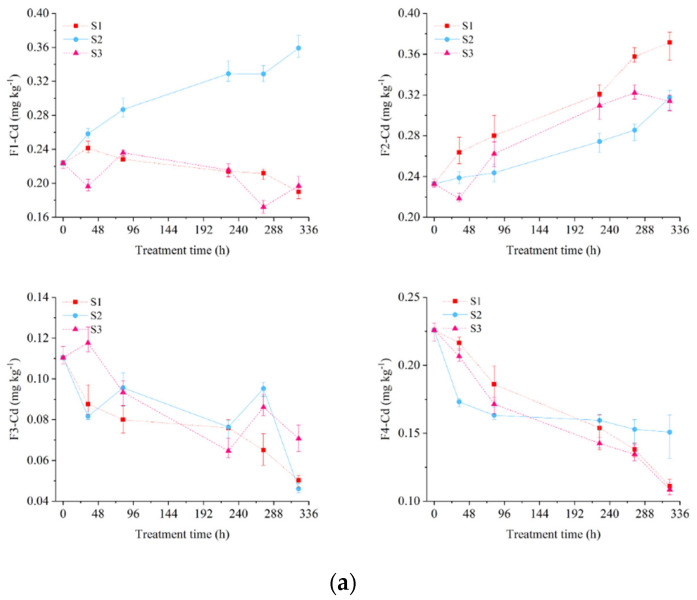

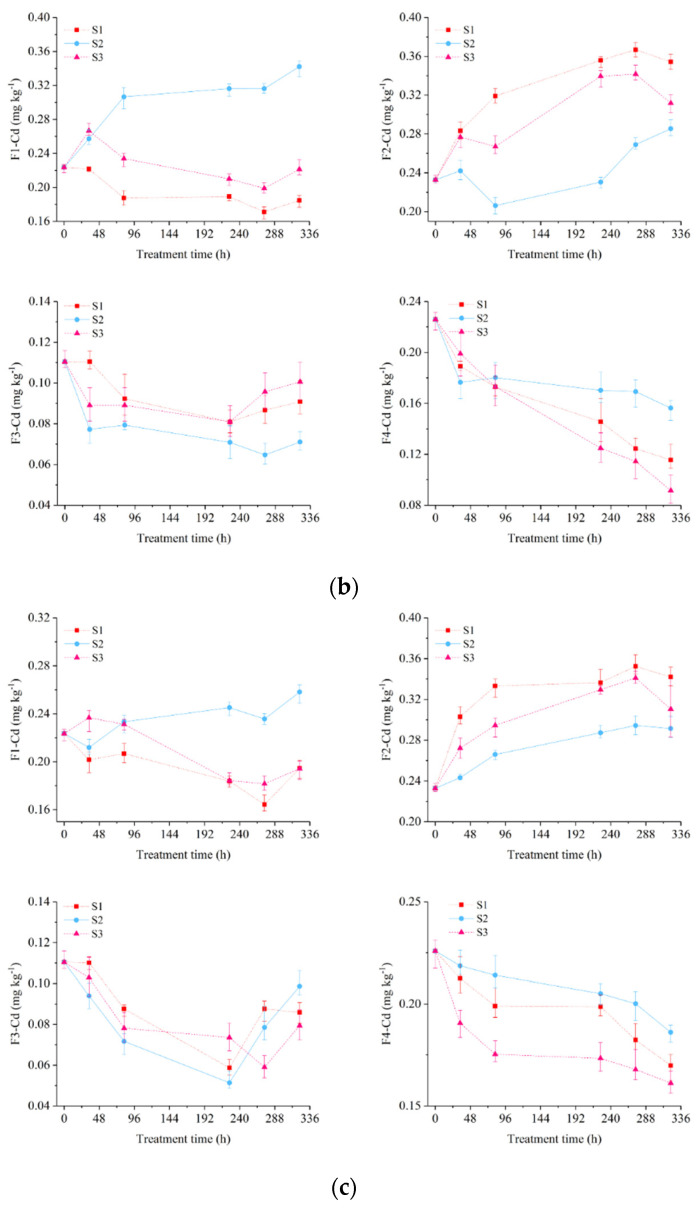
Variation of Cd fraction concentration at EKR 1.0 (**a**), EKR 0.8 (**b**), and EKR 0.5 (**c**), respectively. F1-Cd, F2-Cd, F3-Cd, and F4-Cd were the exchangeable fraction, reducible fraction, oxidizable fraction, and residual, respectively.

Figure 3 showed the migration and redistribution of Cd concentrations in different fractions during the EKR process. Taking EKR 1.0 as an example, the concentrations of F1-Cd in S1 and S3 sections decreased by 15.07% and 11.86%, respectively. The concentration of F1-Cd in S2 section increased by 60.64%. The concentrations of F2-Cd increased by 59.58%, 36.32%, and 34.80%, respectively, in different soil sections. The concentrations of F3-Cd decreased by 54.46%, 58.25%, and 35.99%, respectively, in different soil sections. The concentrations of F4-Cd decreased by 50.84%, 33.22%, and 51.91%, respectively, in different soil sections. Similar results were also observed for EKR 0.8 and EKR 0.5 treatments. Finally, for all three treatments, the concentrations of F1-Cd decreased in the S1 and S3 sections, and increased in the S2 section. The concentrations of F2-Cd increased in all three sections. The concentrations of F3-Cd and F4-Cd decreased in all three sections. The magnitude of the change in Cd concentrations decreased with the decrease in voltage gradient.

At different voltage gradients, the variation of F3-Cd content during EKR process was irregular, which may be related to the change in soluble organic carbon caused by the change in soil moisture [52]. The concentrations of F4-Cd gradually decreased. The removal rates of F3-Cd and F4-Cd near the electrodes were higher than those in the middle soil section. Correspondingly, the increases in F2-Cd near the electrodes were higher than that in the middle soil section. It indicated that electrolysis reactions led to the transformation of soil Cd from stable state to the labile state under EKR treatment. The concentration of F1-Cd decreased in the S1 and S3 sections, while it increased in the S2 section. This confirmed the promoting effect of repeating polarity reversal on the migration of F1-Cd towards the S2 section. The results showed that soil Cd desorbed from oxidizable and residual fractions to the exchangeable and reducible fractions under EKR technology. Polarity reversal significantly promoted the enrichment of exchangeable Cd in the middle soil section.

### 3.3. Speciation of Heavy Metals in the Soil

The mobility and bioavailability of heavy metals were related to their chemical forms in the soils. The exchangeable, reducible, oxidizable, and residual fractions of Cd in the initial soil were 28.20%, 29.37%, 13.93%, and 28.50%, respectively. Traditionally, the exchangeable and reducible fractions (F1-Cd and F2-Cd) were considered as the labile and easily available parts in soil [53]. Metals found in these two fractions were considered as the electromigration-accessible phases [54]. In this study, the electromigration-accessible phase of Cd was 57.57%. Therefore, Cd was expected to migrate efficiently during EKR process.

**Figure 4:**
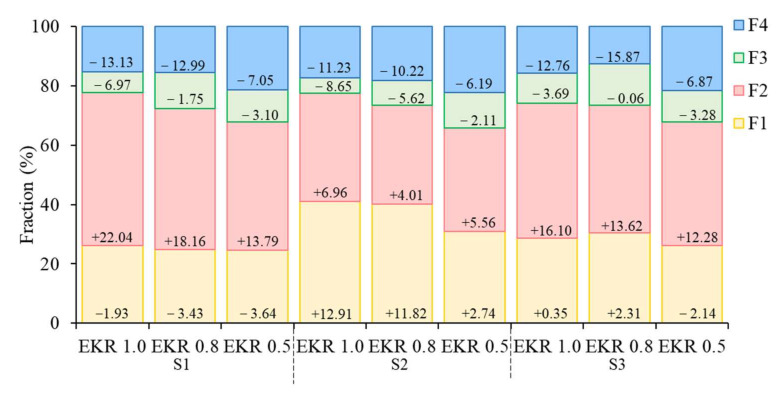
Cd fractions (%) at the end of EKR process. Figures in the graph indicated the variations of different fractions of Cd compared to the initial one. “+” indicates increase in the percentage, and “−” indicates the decrease in the percentage. EKR indicates electrokinetic remediation. F1-Cd, F2-Cd, F3-Cd, and F4-Cd indicate the exchangeable fraction, reducible fraction, oxidizable fraction, and residual, respectively.

Figure 4 showed Cd fractions of different soil sections at the end of EKR process. Figures in the graph indicated the changes in different fractions of Cd compared with the initial one. The “+” indicates the increases in the percentages, and the “−” indicates the decreases in the percentages. At the end of EKR process, the F2-Cd fractions increased, and the F3-Cd and F4-Cd fractions decreased in different soil sections at different voltage gradients. The F1-Cd fractions slightly decreased in the S1 section, and significantly increased in the S2 section at different voltage gradient. However, the variations of F1-Cd did not show a consistent pattern in S3 section. The results of heavy metals speciation analysis contributed to the understanding of the mechanisms of EKR with polarity reversal. It indicated that the oxidizable and residual phases were desorbed from the soil, and were dissolved to the reducible and exchangeable phases. Finally, the exchangeable phases were gathered in the S2 section by repeating the polarity reversal operations.

### 3.4. Changes in Total Cd during EKR process

**Figure 5:**
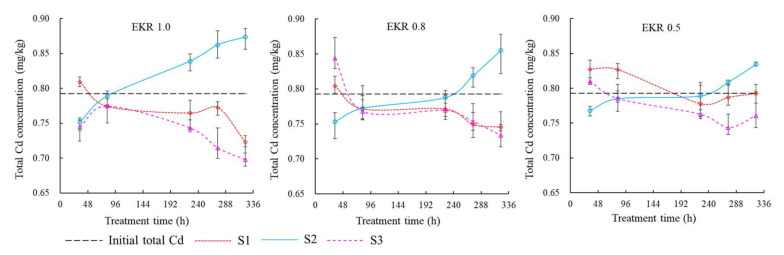
Total Cd concentration variation under different voltage gradients.

Figure 5 showed changes in total Cd concentration during EKR process. In general, the mobilization and transformation of Cd seemed to be similar at different voltage gradients. It was obvious that Cd contaminants in the soil migrated from the S1 and S3 sections to the S2 section. At 34 h, Cd contents in the S1 and S3 sections were higher than the initial values. Cd content showed a slight increase from 274 h to 322 h under the EKR 0.5 treatment. The phenomena may be related to the different lengths of the path for Cd migration under different soil moistures. Uneven sampling caused by the U-shaped shovel—instead of sampling whole soil sections—may be another reason. Generally, the slopes of the lines from 96 h to 226 h were smaller compared with the other time periods, indicating more moderate variations in Cd concentrations. It was related to the lower soil moisture content and smaller electric current during this time period. The moisture content decreased along with time until it dropped to 0.301 g g^−1^. Then, the soil was irrigated to saturation at 154 h (Figure 2). In Figure 5, the slopes of the lines continued to increase after 226 h. The variation of Cd concentrations had a negative relationship with the soil moisture content. At lower soil moisture content, the current decreased, and the migration and transformation efficiency of the Cd contaminants was reduced. The above results further indicated that, when the soil gravimetric moisture content was higher than about 0.35 g g^−1^ (Figure 2), the current was higher, and the metal ions could have a desirable migration efficiency. Briefly, when applying the EKR technology with polarity reversal operation in paddy fields under CI, soil gravimetric moisture content should be kept higher than 0.35 g g^−1^, or should be powered when the moisture content was above 0.35 g g^−1^ to achieve a reasonable performance.

Cd contaminants eventually gathered in the S2 section. The total Cd contents increased by 10.19%, 7.85%, and 5.27%, respectively, for EKR 1.0, EKR 0.8, and EKR 0.5. Repeated polarity reversal operation at a higher voltage gradient could effectively mobilize the contaminants towards the S2 section. At the end of EKR experiment, in the S1 and S3 sections, the total Cd decreased by 8.83% and 12.93%, respectively, for EKR 1.0; the total Cd decreased by 5.97% and 8.51%, respectively, for EKR 0.8; the total Cd decreased by 0.04% and 5.96%, respectively, for EKR 0.5. The removal of Cd contaminants was asymmetric between the S1 and S3 soil sections. In previous studies, the lower pH formed by the acid front promoted the dissolution of heavy metals. It was essential for the efficient remediation of contaminated soil [50]. In this study, due to the neutral soil pH, the migration rate of Cd contaminants was at a low degree even at higher voltages. After 14 d of remediation, the Cd contents in all three soil sections were higher than the risk screening value of 0.6 mg kg^−1^.

### 3.5. Citric Acid Preacidification Enhancement EKR Technology

In order to improve the migration rate, citric acid was added into the contaminated soils as an enhancement solution. The citric acid preacidification enhancement experiment was carried out over 6 d. Table 2 illustrated the current intensity, total Cd concentration, and soil pH variation after EKR^C^ experiments. The current intensity gradually decreased as the soil became dry. Compared with the results in Figure 2a, citric acid resulted in a significant increase in current intensity. Soil pH ranged from 6.19 to 6.65 following citric acid preacidification. The soil with citric acid was slightly acidic. It was related to the buffering capacity of soil and biodegradability of citric acid. There were no significant differences in soil pH among treatments. This was consistent with the results in Figure 2. The electromigration of heavy metal ions in soil between anode and cathode was the key process investigated in this study. Compared with the EKR experiment, citric acid had a significant contribution to the migration of Cd in the EKR^C^ experiment. As can be seen in Table 2, polarity reversal also significantly promoted the enrichment of F1-Cd in S2 section, while soil Cd was gathered in the middle soil section.

According to Equation (1), the total Cd increased by 45.16%, 32.24%, and 21.10%, respectively, for EKR 1.0, EKR 0.8, and EKR 0.5. At the end of EKR experiment, in S1 and S3 sections, the total Cd decreased by 24.70% and 26.61%, respectively, for EKR 1.0; decreased by 16.45% and 18.93%, respectively, for EKR 0.8; and decreased by 8.64% and 15.80%, respectively, for EKR 0.5. The results evidenced that citric acid enhancement experiment significantly improved the migration of metals in a shorter time. The total Cd migration in the S1 and S2 sections was almost 2.5 times higher than that in the unenhanced EKR experiments (except for S3 section under EKR 0.5 treatment). Total Cd migration in the S2 section was almost four times higher than that in the unenhanced EKR experiments. The solubility and mobility of Cd ions were greatly enhanced following the EKR^C^ progress. With a higher voltage gradient, the migration rate of Cd increased significantly. At the end of the EKR^C^ experiment, the Cd concentrations in the S1 and S3 sections were lower than 0.6 mg kg^−1^ at 1.0 V/cm voltage gradient, which met the requirements of the Chinese soil environmental quality standards.

The increase in Cd in S2 section was used as the target to analyze the electrical energy consumption per unit mass (Table 3). The energy consumption of EKR^C^ 1.0 was 0.400 kWh kg^−1^, of EKR^C^ 0.8 was 0.360 kWh kg^−1^, and of EKR^C^ 0.5 was 0.230 kWh kg^−1^. In general, low voltage or electric current led to low energy consumption. However, the operating time was an important parameter, as it significantly influences decontamination efficiency and energy consumption. The shorter the operation time, the smaller the energy consumption [55]. Although EKR^C^ 0.5 had the lowest energy consumption, the migration rate was too low to achieve the remediation of contaminated soil. Thus, the voltage gradient of 0.5 V/cm was not recommended as a solution. The energy consumption of EKR^C^ 1.0 was slightly higher than that of EKR^C^ 0.8. The results were generally satisfactory regarding the migration efficiency and the energy consumption at the two voltage gradients. In the future, the voltage gradient chosen for in situ remediation will depend on the distance between the anode and the cathode. For the larger distance between the electrode plates, the lower voltage gradient of 0.8 V/cm can be recommended for safety reasons [56]. If the distance between the electrode plates is around 27 cm, then the voltage gradient of 1.0 V/cm can be applied for the shorter time and the higher efficiency of remediation.

## 4. Discussion

### 4.1. Mechanisms and Functions of EKR with Polarity Reversal

The transport mechanisms of heavy metal pollutants between the electrodes include electromigration, electroosmosis, and electrophoresis, coupled with electrolysis reactions [57]. Electromigration is the movement of charged ions in soil under the pulsed electric fields. It is the dominant transport mechanism of inorganic contaminants. Metal cations move toward the cathode and anions toward the anode. The electromigration velocity ($V_{em}$) is related to the electric gradient (*E*), ionic valence (*z*), the velocity of ionic mobility ($u_{i}$), and the Ferrari constant (*F*).

(3) $$V_{em}=u_{i}zFE$$

Electroosmosis is the movement of soil pore fluid under the pulsed electric fields. Soil pore liquid carried out movement relative to the charged soil surface, and its movement direction is the same as the cation migration. Electrophoresis is the movement of charged partials or colloids under the pulsed electric fields. It has little effect on the movement of inorganic contaminants, and can be negligible in the EKR process.

During the EKR process, the electrolysis of water is an important reaction. As shown in Equation (4), the oxidation of water releases H^+^ ions at the anode zone, which contributes to desorb heavy metals from the soil. The reduction reaction of water releases OH^−^ ions at the cathode zone, which leads to the precipitation of heavy metals.

(4) $$\begin{array}{c} \mathrm{Anode}:\;2H_{2}O-4e^{-}\to 4H^{+}+O_{2}↑\\ \mathrm{Cathode}:\;2H_{2}O+2e^{-}\to 2{\mathrm{OH}}^{-}+H_{2}↑ \end{array}$$

**Figure 6:**
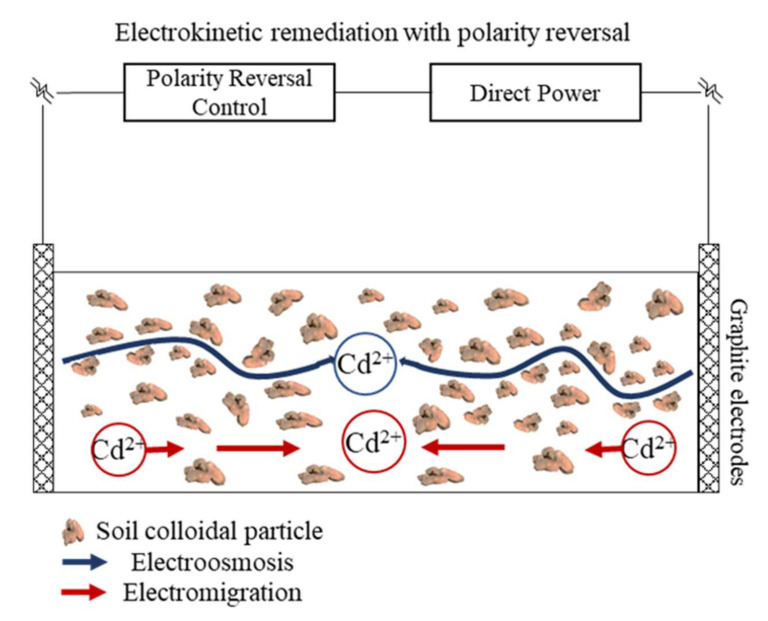
Transport mechanisms of Cd contaminants in the soil under EKR with polarity reversal.

An electric field with polarity reversal was applied in this study. The anode and cathode were continuously exchanged during the EKR process. The H^+^ ions produced by electrolysis of water contributed to the desorption of Cd from the soil surface and released them into the soil liquid phase as free-moving ions, which increased the number of mobile ions in the EKR system. The ions were constantly migrating through the transport mechanisms of electromigration and electroosmosis. Cd ions showed reciprocal motion between cathode and anode. They migrated and accumulated in the S2 section. The Cd migration rate decreased with decreasing voltage gradient. On the other hand, the electric field with polarity reversal avoided the enrichment of H^+^ and OH^−^ ions in the soil, so that the soil zeta potential was not reduced, which facilitated the electroosmosis. The electrolysis of water was more intense, and the electroosmotic flow was higher, near the electrodes. On the contrary, the electrolysis of water was weaker in the S2 section away from the electrodes. Driven by transport mechanisms of electroosmotic, Cd ions advance from the area around the electrodes toward the S2 section. As shown in Figure 6, an electric field with polarity reversal helped to control the migration direction of Cd, thus allowing Cd to migrate directionally to specific areas.

### 4.2. Effects of Soil Moisture on Electrokinetic Remediation

The thermal effect, as the energy losses accompanying the work carried out by electrical energy, is the most common mechanism. Ho et al. found that thermal effect due to temperature rise was not significant in bench-scale studies (less than 1 m in length) [58]. However, in field plot experiments, the voltage gradient more than 1 V/cm led to considerable rise in the soil temperature which may not be neglected [59]. Saline–sodic soils were associated with high pH > 8.2. These properties made considerable rise in the soil temperature which may not be neglected during EKR process [60]. In this study, the differences in soil moisture contents among different voltage gradients were negligible (Figure 2). It meant that the higher voltage gradient did not have significant impact on the soil temperature and moisture. The reasons were mainly due to the bench-scale study and the neutral soil pH.

It was found that high soil moisture could increase the current density to accelerate Cd contaminants migration. The lower limit of soil moisture for EKR was 0.35 g g^−1^. Previous EKR studies have also indicated that the soil moisture influenced the dissolution rate and migration efficient of soil-mobilized Cd. Higher soil moisture enhanced the dissolved contaminant transport by ionic migration and electroosmosis, and hence improved removal efficiency [60]. Soil moisture complementation improved the diffusion and migration of Cd [61]. It can be assumed that higher soil moisture strengthened the current density, and had a positive effect on Cd migration.

### 4.3. In Situ Electrokinetic-Assisted Phytoremediation Technology

EKR technology has been developed for remediating contaminated soil. In majority cases, it was proposed that the electrodes inserted into the electrolyte solutions, attached to the contaminated soils. Thus, the pollutants can be removed from the cathode [43,62]. However, setting up electrolyte cells for field applications can increase costs and operational difficulties. In this study, the electrodes were inserted vertically into the soils. EKR with polarity reversal was applied to achieve the migration and redistribution of Cd in the contaminated soil between the electrode plates. Before reversing the polarity, the S1 section was the anode where the oxidation of water produced H^+^ ions. After reversing the polarity, the former cathode (S3 section) would be the new anode, and Cd ions would migrate towards the new cathode. It had limited adverse effects on soil pH. The purpose of this study was to drive the enrichment of Cd contaminants to specific sites. The electromigration was the major mechanisms driving the movement of metals. Thus, EKR with polarity reversal were not limited to a single soil. It was also acceptable for both saturated and unsaturated soils and may have a wider significance.

**Figure 7:**
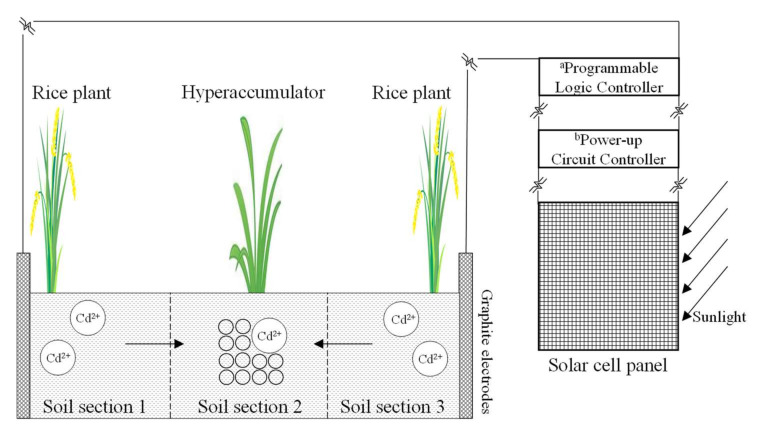
The electrokinetic-assisted phytoremediation technology of Cd-contaminated soil remediation during rice production process. ^a^ Programmable logic controller: with the functions of timing control and action sequence control, it can be programmed to automatically control pulsed electric fields and polarity exchanging operation. ^b^ Power-up circuit controller: boosting the output voltage of solar cell to meet the needs of field experiments.

Based on the above results, an innovative electrokinetic-assisted phytoremediation technology can be proposed. Phytoremediation was limited in practical applications due to its low efficiency. The pulsed electric fields could enhance the desorption of heavy metal contaminants from soil, and significantly improve the remediation efficiency. In recent years, the electrokinetic-assisted phytoremediation technology has been developed. By applying DC electric field, heavy metals were redistributed from anode to cathode in the soil. There was an enhancement of metal hyperaccumulator uptake near the cathode [63,64,65]. As mentioned in this study, the exchangeable phase of Cd was significantly enriched in the intermediate, where the hyperaccumulator could be planted to absorb the exchangeable Cd. A large number of hyperaccumulator species are available, such as lantana plant, sedum plumbizincicola, bulrush, and so on [6,66]. Based on the above, we can put forward a scheme of Cd-contaminated soil remediation during rice production process (Figure 7). The combined remediation technology can be conducted in paddy fields under CI irrigation [39]. As can be seen in Figure 7, in the field-scale application, the rice crop is planted near the electrodes, and hyperaccumulator is planted between two rows of rice plants. Before planting the rice, citric acid is added evenly into the surface soils at a concentration of 0.1 mol L^−1^ to improve metal solubility. EKR technology with polarity reversal should be conducted at soil gravimetric moisture content ranging from 0.35 g g^−1^ to full saturation to obtain a more desirable efficiency. The pulsed electric fields mimic the use of solar energy as a power source. The remediation was obtained during daytime and stopped through the nighttime. When using solar energy a power source, the power-up circuit controller is used to boost the output voltage of the solar cell to meet the needs of the field experiments. The programmable logic controller can be programmed to automatically control pulsed electric fields and polarity reversal operations. The electrokinetic remediation runs in the daytime and stops at nighttime. The redistribution of Cd contaminants in soil can be expected. Cd contaminants migrate towards the soil section that between two rows of rice plants, where hyperaccumulator plants will take up more Cd contaminants. On the other hand, the removal of Cd and the low toxicity of the residue was achieved in soils close to the electrodes. Soil sections close to the electrodes were suitable for rice growth to avoid absorbing too much Cd contaminants.

Electrokinetic-assisted phytoremediation is a promising technology for soil environmental remediation. The significance of this study was to provide a theoretical basis. The combined remediation technology can be conducted during the rice production process. The methodology ensures the synchronization of purification of contaminated soil and safe production of rice crops. Briefly, the results of this paper provided strong theoretical supports for in situ electrokinetic remediation. It may offer a safe and cost-effective decontamination technique for Cd-contaminated soil. In the future, scaling up the EKR technique with polarity reversal, for field-scale applications, deserves further exploration.

## 5. Conclusions

The results showed that the application of electric fields with polarity reversal caused the migration of Cd contaminants from both ends of the soil compartment toward the intermediary section. In particular, the exchangeable phase of Cd was gathered in the middle area. Power should be supplied at a moisture content above 0.35 g g^−1^ to achieve reasonable performance. Preacidification of the soil with citric acid is an advisable enhancement strategy. The migration rate of Cd was proportional to voltage gradient. The application of citric acid at the voltage gradient of 1.0 V/cm or 0.8 V/cm appeared to be an advisable solution. The results provided strong theoretical supports for in situ electrokinetic remediation. The strategy is suitable to the remediation of mild-Cd-contaminated soil in paddy fields under control irrigation. Rice crop is planted near the electrodes to minimize the Cd contamination in grains. A hyperaccumulator can be planted between two rows of rice to absorb Cd contaminants. The proposed method provides a safe and cost-effective decontamination technique.

## Figures and Tables

**Table 1:** Summary of experimental conditions.

Treatment	Voltage Gradient (V cm^−1^)	Duration (d)	Periodic Power/Day	Polarity Reversal Frequency (h)	Soil Moisture Alternate Wetting and Drying (AWD) Cycles
EKR 1.0	1.0	14	10 h ON/14 h OFF	24	Two AWD cycles
EKR 0.8	0.8				
EKR 0.5	0.5				
EKR^C^ 1.0	1.0	6	10 h ON/14 h OFF	24	One AWD cycle
EKR^C^ 0.8	0.8				
EKR^C^ 0.5	0.5				

**Table 2:** The current intensity, soil pH, and total Cd concentration after the citric acid preacidification enhancement experiment.

Soil Section	Treatment	Intensity Variation (mA)	Finial pH	Final F1-Cd Concentration (mg kg^−1^)	Finial Total Cd Concentration (mg kg^−1^)
S1	EKR^C^ 1.0	459~807	6.25 ± 0.065 ab	0.195 ± 0.022 a	0.597 ± 0.015 c
	EKR^C^ 0.8		6.19 ± 0.040 b	0.183 ± 0.012 a	0.662 ± 0.015 b
	EKR^C^ 0.5		6.33 ± 0.035 a	0.180 ± 0.014 a	0.724 ± 0.011 a
S2	EKR^C^ 1.0	368~647	6.50 ± 0.050 a	0.490 ± 0.019 a	1.151 ± 0.020 a
	EKR^C^ 0.8		6.51 ± 0.080 a	0.421 ± 0.019 b	1.048 ± 0.011 b
	EKR^C^ 0.5		6.65 ± 0.140 a	0.332 ± 0.012 c	0.960 ± 0.018 c
S3	EKR^C^ 1.0	241~426	6.21 ± 0.087 a	0.196 ± 0.009 a	0.582 ± 0.010 b
	EKR^C^ 0.8		6.34 ± 0.087 a	0.192 ± 0.015 a	0.643 ± 0.018 a
	EKR^C^ 0.5		6.21 ± 0.070 a	0.194 ± 0.022 a	0.667 ± 0.016 a

Note: Different letters represent significant differences among treatments at the *p* < 0.05 level (*n* = 3).

**Table 3:** The electrical energy consumption per unit mass ($E_{U}$) for EKR^C^ experiment.

Treatment	the Mass of Increased Cd in S2 Section (kg)	$E_{U}\;(\mathrm{kWh}\;\mathrm{kg}{-}^{1})$
EKR^C^ 1.0	2.51 × 10^−3^	0.400
EKR^C^ 0.8	1.79 × 10^−3^	0.360
EKR^C^ 0.5	1.17 × 10^−3^	0.230

## Data Availability

The datasets are available from the corresponding author on reasonable request.
